#  Enterovirus A71 Subgenotype B5, France, 2013

**DOI:** 10.3201/eid2104.141093

**Published:** 2015-04

**Authors:** Audrey Mirand, Lucie Molet, Chervin Hassel, Hélène Peigue-Lafeuille, Flore Rozenberg, Jean-Luc Bailly, Cécile Henquell

**Affiliations:** Clermont Université, Clermont-Ferrand, France (A. Mirand, C. Hassel, H. Peigue-Lafeuille, J.-L. Bailly, C. Henquell);; Centre National de Référence des Entérovirus-Parechovirus, Clermont-Ferrand (A. Mirand, H. Peigue-Lafeuille, J.-L. Bailly, C. Henquell);; Hôpital Cochin, Paris, France (L. Molet, F. Rozenberg);; Université Paris Descartes, Paris (L. Molet, F. Rozenberg)

**Keywords:** Enterovirus A71, hand, foot and mouth disease, neonatal enterovirus infection, subgenotype B5, viruses, France, *Suggested citation for this article*: Mirand A, Molet L, Hassel C, Peigue-Lafeuille H, Rozenberg F, Bailly JL, et al. Enterovirus A71 subgenotype B5, France, 2013 [letter]. Emerg Infect Dis. 2015 Apr [*date cited*]. http://dx.doi.org/10.3201/eid2104.141093

**To the Editor:** We report the detection of human enterovirus A71 (EV-A71) subgenotype B5 in France, 6 years after it was first detected in Europe. EV-A71 belongs to the *Enterovirus A* species (genus *Enterovirus*, family *Picornaviridae*) and is a major cause of hand, foot and mouth disease (HFMD), sometimes associated with severe neurologic complications (*1*)*.* EV-A71 strains are classified in 6 genotypes, A–F, (*2*) but most of the circulating strains belong to genotypes B and C and to 11 subgenotypes (B0−B5, C1−C5) (*1*). Genotypes B and C have been reported in HFMD epidemics in the Asia-Pacific region; different subgenotypes cause nationwide epidemics that usually occur every 2−3 years (*1*). During 1963−1986 in Europe, Australia, and the United States, enterovirus infections were caused by viruses from subgenotypes B0, B1, and B2, but since 2000, infections with C1 and C2 viruses have begun to predominate (*3**,**4*). The other subgenotypes have been reported rarely in Europe; the C4b and C4a strains were identified in France, Germany, Austria, and Denmark in 2004 and 2012, respectively (*4**–**6*), and the B5 subgenotype was reported in Denmark in 2007 (*7*).

In November 2013, a 3-week-old boy was admitted to the emergency unit of a hospital in Compiègne, France, with a 48-hour history of fever and irritability. He was born at term after an uneventful pregnancy and delivery. On admission, he had normal vital signs. He had had contact with a cousin with oral ulcerations, but no information was available about the source of the cousin’s infection.

Laboratory testing revealed moderate cytolytic hepatitis. Complete blood count results were within reference values. Cerebrospinal fluid showed pleiocytosis (38 leukocytes, 81% polymorphonuclear cells) with protein and glucose levels with reference ranges. Bacterial cultures of blood and cerebrospinal fluid were negative. Enterovirus genome was detected in serum samples and cerebrospinal fluid by reverse transcription PCR. The infant made a steady recovery and was discharged 10 days after admission, with no apparent adverse outcome. Final diagnosis was neonatal enterovirus infection with meningitis.

Genotyping was performed on the serum specimen by using seminested reverse transcription PCR amplification and sequencing of the viral protein 1 gene. Phylogenetic investigation with sequences of reference strains representing all subgenotypes indicated that the isolate from the patient, designated PAR024102_FRA13, belonged to the EV-A71 B5 subgenotype. We investigated the putative origin of the strain by comparing 248 nonredundant complete 1D sequences of EV-A71 B5 strains following a Bayesian phylogenetic approach. PAR024103_FRA13 shared a most recent common ancestor (posterior probability = 1) with virus strains sampled in China in 2009 and in Taiwan during the 2011–2012 outbreak, (*8*) but was only distantly related to them (data not shown).

Further analyses with 274 partial 1D gene sequences from GenBank (on March 19, 2014) indicated close genetic relationships (posterior probability = 1) with strains isolated in Thailand in 2012 (*9*) (Figure). The complete genome of PAR024103_FRA13 was determined by nucleotide sequencing of 4 overlapping segments obtained by gene amplification (GenBank accession no. LK985324). Sequence comparisons were performed with 13 available EV-A71 B5 complete genomes; the virus strain isolated in France exhibited 92%−99.5% nt similarity (98.9%−99.4% aa similarity) throughout the genome.

**Figure:**
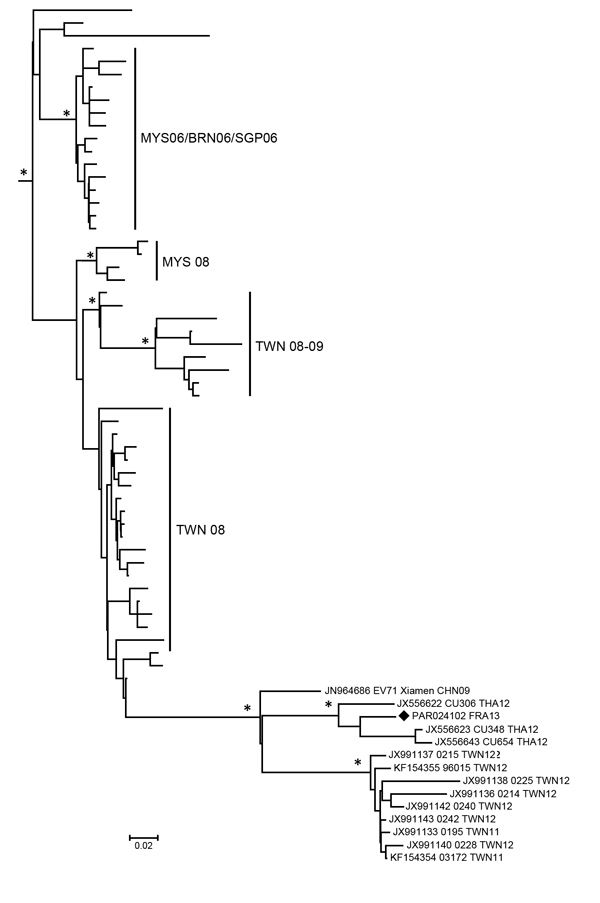
Phylogeny of enterovirus A71 (EV-A71) subgenogroups B4 and B5 inferred with 274 partial 1D gene sequences, France. Black diamond indicates strain PAR024103_FRA13 from this study. The phylogenetic relationships were inferred following a Bayesian method by using a relaxed molecular clock model with an uncorrelated exponential distribution of evolution rates estimated with a general time reversible substitution model and a Bayesian skyline plot as a population model (BEAST version 1.7.5; http://beast.bio.ed.ac.uk/). Sequences were 319 bp. For clarity, only a subtree of EV-A71 B5 sequences is shown, and taxon names are not included. Asterisks indicate nodes with posterior probability density values >0.90. Geographic origins and time of isolation of strains are indicated by the International Organization of Standardization code abbreviation followed by the year of isolation. Scale bar indicates nucleotide substitutions per site.

The EV-A71 B5 subgenotype was first detected in 1999 in Malaysia and spread to several other countries in Asia during the 2000s. Outbreaks causing severe illness and deaths were reported in Japan (2003), Brunei (2006), and Taiwan (2008 and 2012) (*1**,**8*). The first detection of subgenotype B5 in Europe was associated with a recrudescence of EV-A71 infections associated with meningitis and HFMD in Denmark in 2007 (*7*). The overall phylogenetic data are consistent with an introduction of EV-A71 B5 in France by importation of a strain from Asia, possibly from Thailand. Transmission of EV-A71 strains has been shown to occur in Europe as discrete and temporally defined virus introductions, occasionally followed by sustained dissemination (C. Hassel, unpub. data).

The emergence of the Asiatic lineage EV-A71 C4a in Denmark in 2012 is a recent event (*6*). The reemergence of EV-A71 subgenotype B5 in 2008 in Taiwan resulted in the largest outbreak of EV-A71 infection in the past 11 years (*8*). To our knowledge, the B5 subgenotype has not previously been detected in Europe. Global herd immunity produced by circulation of the C2 genotype may protect the European population from the spread of other subgenotypes (*3*). However, given that most countries in Europe do not perform specific surveillance for HFMD and most enterovirus infections are asymptomatic, this particular subgenotype could be circulating more widely without detection.

Enterovirus infections in neonates and infants are a frequent cause of hospitalization, which may contribute to EV-A71 detection (*5*). However, the development of a national syndromic surveillance targeting HFMD would enable early detection of HFMD outbreaks and any new EV-A71 subgenotype. Attention should also be paid to the potential risks of epidemic spread of EV-A71 outside Asia posed by international travelers.
